# Dissecting the Transcriptional Control of Body Patterning

**DOI:** 10.1371/journal.pbio.0020319

**Published:** 2004-08-31

To build the complex body plan of higher organisms, thousands of genes must act in a coordinated fashion, becoming active at the right time and in the right place to define structures like head, thorax, and abdomen, or cell types like skin, muscle, and bone. One of the central questions for developmental biologists is how such specific spatiotemporal expression of genes is achieved.

The general mechanism of the control of gene expression is well understood: Special proteins, called transcription factors, bind to short stretches of DNA near a gene. By docking to such binding sites, they activate or repress the transcription of the gene into mRNA (which is then translated into protein). Transcription factors often act in a combinatorial fashion—that is, several different factors have to bind in close proximity to each other to achieve a particular transcriptional outcome. As a consequence, their binding sites form clusters, called regulatory elements or modules.

In many contexts, the genes that are activated or repressed encode transcription factors themselves, forming a cascade of transcriptional control events. One such transcriptional control hierarchy is the segmentation gene network in the fruitfly Drosophila. Organized in four tiers and acting in combinatorial fashion, the segmentation genes lay out the anterior-posterior axis of the embryo. In a stepwise refinement of expression patterns, they translate broad, overlapping gradients formed by maternally provided transcription factors into a periodic pattern of 14 discrete stripes that prefigure the 14 segments of the larva. The segmentation gene network has long been one of the prime paradigms for studying transcriptional control, and many researchers have worked over the years to experimentally dissect the regulatory interactions within the hierarchy. For some of the most important genes, the regulatory elements driving their expression and the favored binding sites have been identified. Nevertheless, the picture of transcriptional regulation within the segmentation gene network has remained incomplete.

This is where the research reported by Mark Schroeder et al. comes in: With the sequence of entire genomes available, it's possible to use existing binding site information to computationally search the neighborhood of genes for regulatory elements. The difficulty here is that in higher organisms such as Drosophila, the binding sites are typically short and variable, and the search space is large; on the other hand, the fact that sites cluster—where transcription factors work in concert—aids the task.

To identify regulatory elements, the researchers developed an algorithm, named Ahab, that models the behavior of multiple transcription factors competing for binding sites and fine-tunes the search by detecting clusters of weak sites. Using this approach, Schroeder et al. identified 52 regulatory elements within the segmentation gene network, 32 of them novel. The authors tested a large number of the newly identified modules experimentally by placing them in front of reporter genes that reveal where the modules drive expression within the developing fly. They showed that almost all modules faithfully reproduce the expression pattern of the endogenous gene. To better understand the way segmentation gene modules function, the researchers then systematically analyzed their predicted binding site composition. They correlated the composition of modules with the expression they produce and with the distribution of the transcription factors that bind to them. They were thus able to glean basic composition rules and to derive the mode of action for most of the factors, that is, whether they act as activators or as repressors.[Fig pbio-0020319-g001]

**Figure pbio-0020319-g001:**
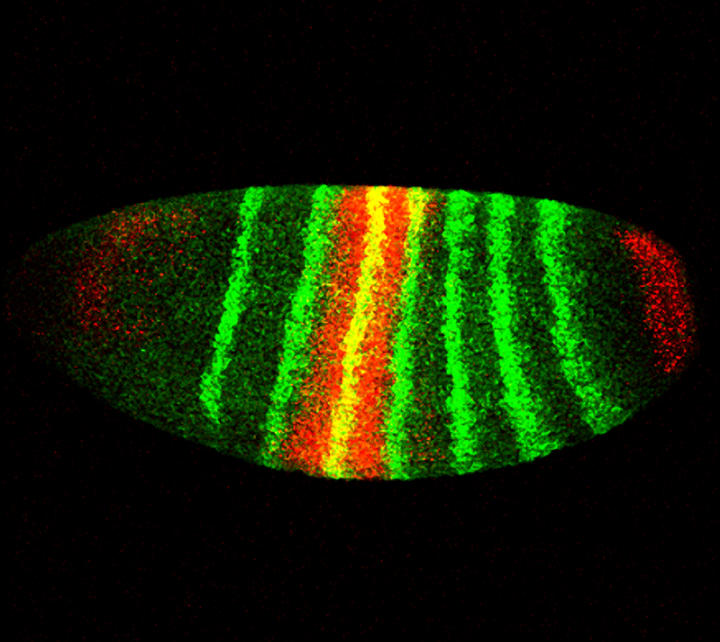
Segmentation in the early Drosophila embryo

Overall, Schroeder et al. show that a computational search can greatly reduce the experimental effort necessary for finding regulatory elements within the genomic sequence. Their study provides an example of how experimental and quantitative methods can be combined to achieve a more global analysis of the regulatory interactions within a transcriptional network.

